# Effect of Different Chemical Surface Treatments on the Shear Bond Strength of Acrylic Teeth With Different High-Impact Denture Base Resins

**DOI:** 10.7759/cureus.42513

**Published:** 2023-07-26

**Authors:** Girija Dodamani, Priyadarshani Pawar, Rohit M Patil, Aamer M Badaam, Saba A Siddiqui, Sneha Mehta

**Affiliations:** 1 Department of Prosthodontics, Jawahar Medical Foundation (JMF) Annasaheb Chudaman Patil Memorial (ACPM) Dental College, Dhule, IND; 2 Department of Prosthodontics, Aditya Dental College, Beed, IND; 3 Department of Orthodontics, Aditya Dental College, Beed, IND

**Keywords:** shear bond strength, bonding, chemical surface treatment, acrylic teeth, denture base

## Abstract

Introduction

The debonding of acrylic teeth from the denture base, particularly in cases of prominent ridges, is a common problem faced by clinicians and patients. The present study was conducted to assess the effects of various chemical treatments on the shear bond strength (SBS) of acrylic teeth bonded to different high-impact denture base materials.

Materials and methods

The present in vitro study was conducted on 80 wax specimens with acrylic teeth bonded to two high-impact denture base materials (DPI Tuff (DPI Dental Products of India Ltd, Mumbai) and Trevalon HI (Trevalon HI, Dentsply, Karnataka)). The two main groups were further divided into four subgroups of 10 specimens each, depending on the chemical treatment at the ridge lap area of the tooth: control group without any chemical treatment, chemical surface treatment (CST) with dichloromethane and monomer mix, CST with ethyl acetate, and CST with acrylic adhesive cyanoacrylate. The SBS was tested using a universal testing machine (UTM). Analysis of variance (ANOVA) and post-hoc Tukey tests were used for statistical analyses.

Results

The mean SBS of Group A (DPI Tuff) was 111.75 N as compared to 118 N in Group B (Trevalon HI). The differences were statistically significant (p<0.05). ANOVA and post-hoc Tukey’s tests revealed significant differences between subgroups. The highest mean SBS was noted with a dichloromethane and monomer mix (1:1 volume), followed by the ethyl acetate, control, and cyanoacrylate subgroups.

Conclusion

The cross-linked acrylic teeth treated with a dichloromethane and monomer mixture (1:1 by volume), processed with Trevalon HI high-impact denture base resin had the highest SBS and thus were indicated for bonding teeth with the suggested denture base.

## Introduction

The loss of natural teeth is a prevalent issue that warrants significant attention, as it necessitates the replacement of the missing teeth in most individuals. Replacement with artificial dentures is the most common treatment preferred by elderly individuals because of their low cost compared to implants and fixed prostheses. Acrylic teeth are usually preferred over porcelain teeth because they are chemically bonded to the denture base material, easily available, easily trimmed, and inexpensive [1]. The introduction of cross-linked acrylic teeth led to a further increase in their stain resistance, fracture, and abrasive resistance but reduced their bond strength with the denture base [2].

Both doctors and patients face difficulties when acrylic teeth debond from the denture base because they need to rebond them mechanically or chemically. It has been estimated that a considerable proportion, ranging from 22% to 30% of repairs for dentures, are associated with the detachment of teeth, primarily in the anterior region. This phenomenon can be attributed to the limited availability of ridge lap surface areas for bonding purposes and the specific orientation of the stresses experienced during functional activities [3].

The mechanical method, such as roughening the surface by sandblasting, does not provide sufficient improvement in the bond strength and is a time-consuming procedure [4]. Chemical methods involve the use of various chemicals such as monomers, non-polymerizable solvents, dissolved poly methyl methacrylate (PMMA), and a combination of the above or adhesives [5]. The chemical formulation of PMMA also has an impact on the bonding between the tooth and denture base [6]. Owing to conflicting evidence regarding the benefits of different chemical surface treatments for teeth, the current study was conducted to compare the effects of surface treatment with different chemical solutions on the bond strength between cross-linked acrylic teeth and various brands of high-impact, heat-cure denture base materials. The null hypothesis of the study was that shear bond strength (SBS) between the tooth and the base would not change after chemical surface treatments.

## Materials and methods

This in vitro study was conducted in the Department of Prosthodontics, ACPM Dental College by a single researcher. Ethical committee approval was not required, as this was an in vitro study, which did not use any human material.

Sample size calculation

The sample size was calculated using GPOWER software (Ver.3.1, Franz Faul University, Keil, Germany) with a type 1 error of 0.05, a beta error of 0.2, and a power of 80%. The sample size was 76. Hence, the study was conducted on 80 samples divided into two main groups of high-impact, heat-cure denture base resin as follows: Group A, DPI Tuff (DPI Dental Products of India Ltd, Mumbai): 40 samples; Group B: Trevalon HI (Trevalon HI, Dentsply, Karnataka): 40 samples. They were further divided into four subgroups each, comprising 10 samples in each subgroup as follows: Subgroup 1, control without any chemical surface treatment (CST); subgroup 2, CST with dichloromethane + monomer mix (1:1 volume); Subgroup 3, CST with ethyl acetate; and Subgroup 4, CST with acrylic adhesive cyanoacrylate. Cross-linked acrylic teeth (TrutonXL upper right central incisors, mold M1, shade 23; Super Dental Products, Wazirpur, Delhi) were used in this study.

Methodology for preparing test specimens

**Figure 1:**
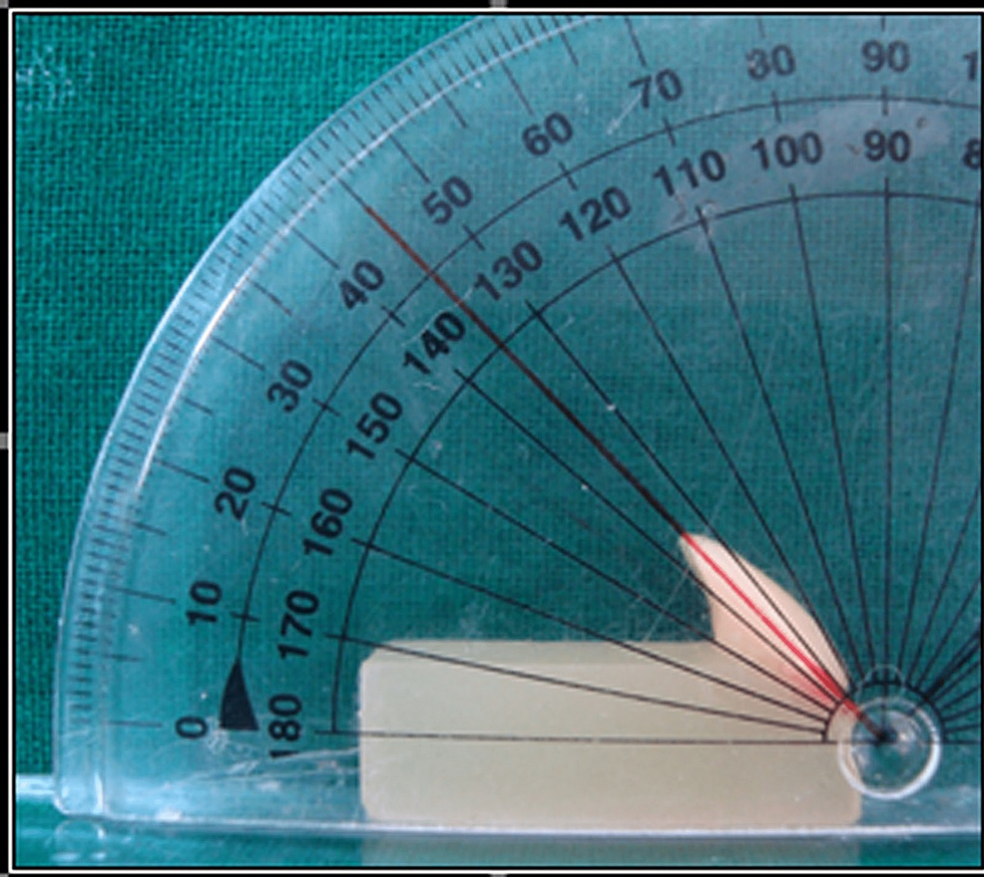
Master wax specimen

The main specimen was made with the acrylic tooth's long axes oriented at a 45-degree angle with regard to the base of an 8 x15x25 cm wax block, where the tooth's ridge lap area made contact with the base, in order to create the mold (Figure 1).

**Figure 2:**
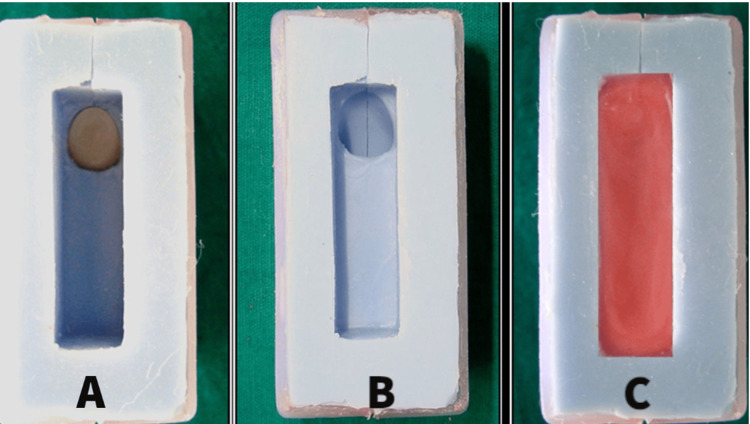
Silicon mold for the preparation of test specimens A: Silicon mold; B: Silicon mold with acrylic tooth; C: Wax-filled mold

A silicone mold was fabricated from this specimen, which was used to form 80 wax specimens by placing the acrylic tooth in it and pouring molten wax over it (Figure 2).

**Figure 3:**
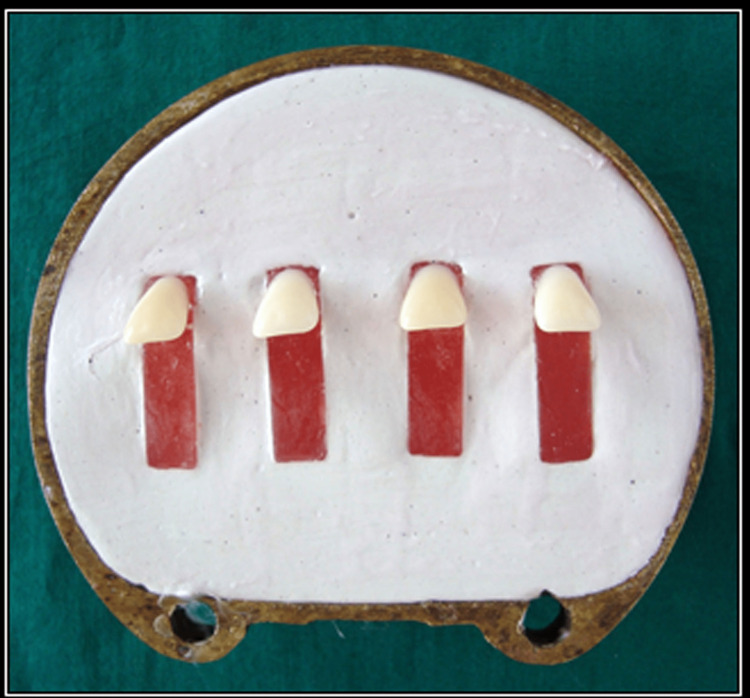
Investment of test specimens

A profile projector was used to validate the angle of the tooth in each block and eliminate any inaccuracies caused by variations in the tooth alignment. The specimens were processed using the conventional heat-curing method (Figure 3) [7].

The chemical surface treatment was performed as follows: Subgroup 2, the ridge lap area was wetted with a mixture of dichloromethane and monomer (1:1 by volume) with a cotton tip applicator and left for 4 min for drying before packing; Subgroup 3, the ridge lap area was wetted with ethyl acetate with a cotton tip applicator, and left for 2 min; and Subgroup 4, the ridge lap area was wetted with a drop of cyanoacrylate adhesive. To simulate in vivo conditions, the test samples were subjected to 6000 cycles of thermocycling in distilled water between 5 °C and 55 °C, with a one-minute dwell time [1].

Testing of shear bond strength (SBS) of the test specimens

**Figure 4:**
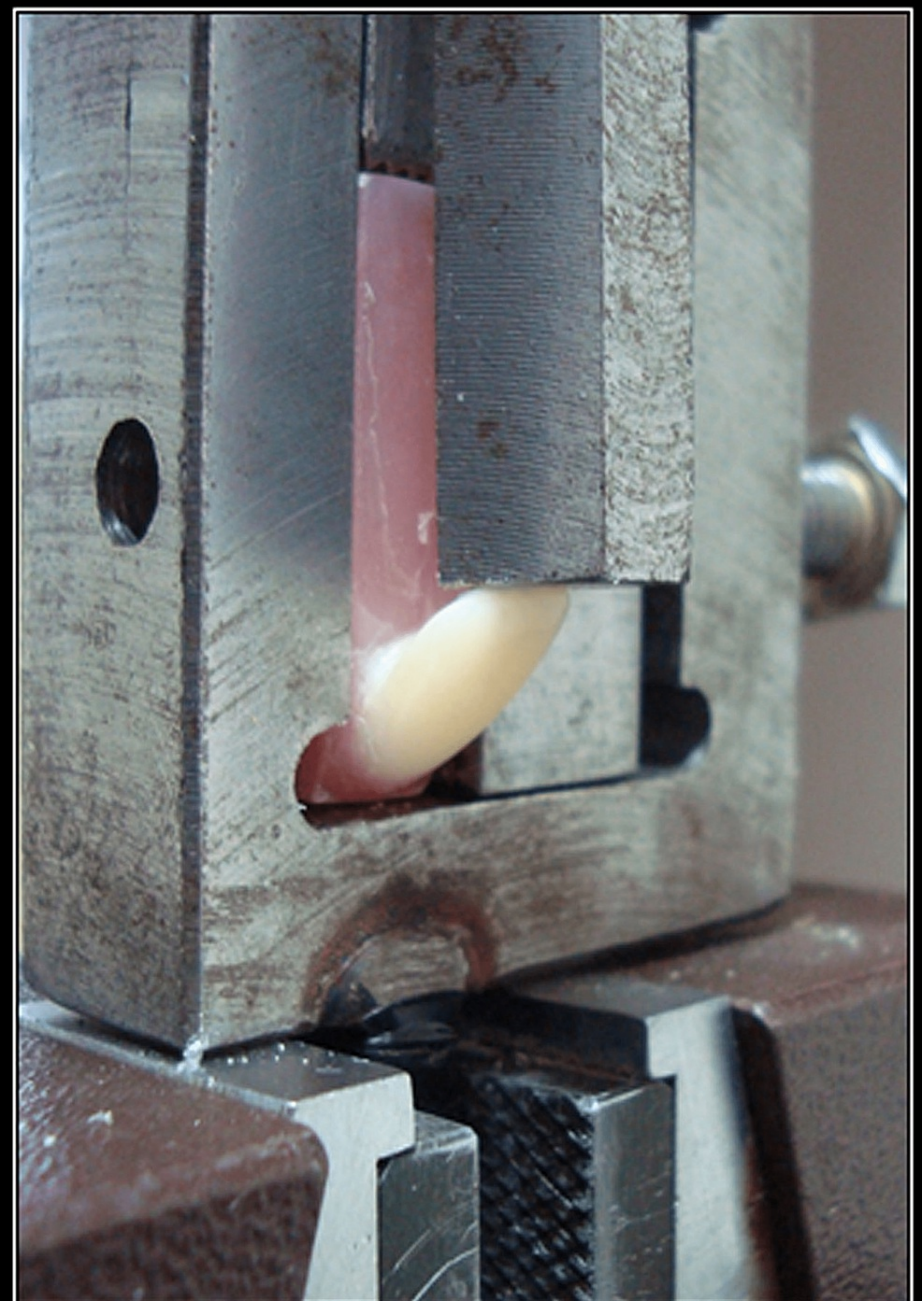
Shear bond strength (SBS) analyzed with the universal testing machine (UTM)

The prepared specimens were subjected to load testing using a universal testing machine (UTI) to apply a load on the incisal edge of the specimen at a crosshead speed of 0.5 mm/min for testing the SBS (Figure 4).

All tests were performed under uniform atmospheric conditions of 200±10 °C and 40 ± 1% relative humidity. The load was applied until the denture teeth were separated from the denture base resin. Upon fracture, the resulting bond strengths were recorded and statistically analyzed.

Statistical analysis

The collected data were entered into Microsoft Excel (Microsoft Corporation, Redmond, WA) and statistically analyzed using SPSS software, version 21 (IBM Corp., Armonk, NY). Descriptive statistics were calculated using means and standard deviations for each group, and a two-way analysis of variance (ANOVA) test was used for inter-group comparisons. This was followed by a post-hoc Tukey test for intra-group comparisons.

## Results

The Shapiro-Wilk test was used to check the normality of the data, and the data were found to be normally distributed. The results of the study revealed that the mean SBS of Group B (Trevalon HI) was significantly higher than that of Group A (DPI Tuff) (Table 1).

**Table 1:** Inter-group comparison of mean SBS in subgroups (SG) of Group A and Group B with independent student t-test p<0.05: statistically significant

Groups	Subgroups (SG)	df	Mean Difference	t-value	p-value
Group A	SG 1 [n=10]	18	4.6	4.1	0.001
Group B	SG 1 [n=10]				
Group A	SG 2 [n=10]	18	6.5	6.9	0.000
Group B	SG 2 [n=10]				
Group A	SG 3 [n=10]	18	4.5	3.3	0.005
Group B	SG 3 [n=10]				
Group A	SG 4 [n=10]	18	10.5	8.8	0.000
Group B	SG 4 [n=10]				

The intergroup comparison of the mean SBS in the subgroups of Groups A and B showed a statistically significant difference (Table 2).

**Table 2:** Comparison of mean shear bond strength (SBS) of Group A (DPI Tuff) and Group B (Trevalon HI) using the independent student t-test p<0.05: statistically significant

Groups	No. of test specimens (n=80)	Mean Shear Bond Strength (N)	df	t-value	p-value
Group A	40	111.7±26.3	1	128	0.000
Group B	40	118.2±24.2			

A one-way ANOVA test for intra-group comparison of the mean SBS of subgroups in Groups A and B revealed a p-value of <0.001, which showed a statistically significant difference in the subgroups (Table 3).

**Table 3:** Intra-group comparison of mean SBS in subgroups (SG) of Group A and Group B using the ANOVA test p<0.05: statistically significant; ANOVA: analysis of variance

Groups	No. of test specimens (n)	Mean SBS in newtons (N) in SG 1 [n=10]	Mean SBS in newtons (N) in SG 2 [n=10]	Mean SBS in newtons (N) in SG 3 [n=10]	Mean SBS in newtons (N) in SG 4 [n=10]	F-value	p-value
Group A	40	118.8±1.45	134.1±1.8	126±2.6	68±1.19	2080	0.000
Group B	40	123.5±2.7	140.6±1.9	130.5±2.7	78.5±3.1	835	0.000

This was further confirmed by post-hoc analysis (Tables 4, 5).

**Table 4:** Intra-group comparison of mean SBS of subgroups in Group A using the post-hoc Tukey test p<0.001: highly significant

Subgroups	Mean difference	F-value	p-value
SG1 vs SG 2	15.25	23.325	p<0.001
SG1 vs SG 3	7.125	10.898	p<0.001
SG1 vs SG 4	50.875	77.814	p<0.001
SG2 vs SG 3	8.125	12.427	p<0.001
SG 2 vs SG 4	66.125	101.139	p<0.001
SG 3 vs SG 4	58	88.712	p<0.001

**Table 5:** Intra-group comparison of mean SBS of subgroups in Group B using post hoc Tukey test p<0.001: Highly significant

Subgroups	Mean difference	F-value	p-value
SG1 vs SG 2	17.125	18.039	p<0.001
SG1 vs SG 3	7	7.374	p<0.001
SG1 vs SG 4	45	47.402	p<0.001
SG2 vs SG 3	10.125	10.665	p<0.001
SG 2 vs SG 4	62.125	65.441	p<0.001
SG 3 vs SG 4	52	54.775	p<0.001

The highest mean SBS was noted with the dichloromethane and monomer mix (1:1 volume) in both groups (134.1±1.8 N in Group A, 140.6±1.9 N in Group B), followed by ethyl acetate (126±2.6 N in Group A, 130.5±2.7 N in Group B), control (118.8±1.45 N in Group A, 123.5±2.7 N in Group B), and the least was seen in the cyanoacrylate subgroup (68±1.19 N in Group A, 78.5±3.1 N in Group B). The mean SBS of all chemical solutions was higher with Trevalon HI than with DPI Tuff high-impact denture base material.

## Discussion

The present study was conducted to assess the effects of different chemical treatments on the SBS of cross-linked acrylic teeth bonded to two high-impact denture base materials. Thermocycling was performed to simulate oral conditions.

In clinical practice, the strength of the bonding between the tooth and denture base depends on a variety of factors such as the adhesion of the parts to one another, the properties and dimensions of the materials, and the mechanical connection between the parts [8].

In the present study, the mean SBS was better with the Trevalon high-impact denture base material than with the DPI Tuff. Our findings were in contrast to those of Gupta et al., where the DPI tuff had a higher transverse strength than the Trevalon HI [9]. The reason for this disparity may be the difference in testing conditions. As stated earlier, the quality of denture base material affects the bond strength of the tooth to the denture base. Acrylic teeth generally bond better with heat-cured denture base resins than with autopolymerizing resins. This might be due to more complete polymerization in heat-cured resins than in self-cure resins [4]. In previous studies, high-impact heat-cured resins have performed better than conventional heat-cured resins [10]. Our findings are in accordance with those of Cunningham et al., and the reason for the increased bond strength of Trevalon HI may be the presence of a greater amount of cross-linking agent in the monomer (ethylene glycol dimethacrylate), leading to an increase in the strength of the formed interwoven polymer network [11].

Chemical bonding of an acrylic tooth with a denture base requires proper physical contact of the acrylic tooth with the denture base and complete crosslinking between the polymer structures of both [4]. Several methods have been employed to enhance the bonding of denture teeth with a base such as mechanical methods (air abrasion, sandblasting, grinding, and retention grooves). However, owing to the varying results obtained using these procedures, chemical methods have been used [5,8,12].

The null hypothesis was rejected in our study significant difference in shear bond strength was noted after chemical surface treatment. The present study showed the highest SBS with dichloromethane and monomer mix (1:1 volume), followed by ethyl acetate, the control, and the cyanoacrylate subgroups. Dichloromethane and ethyl acetate are volatile, non-polymerizable solvents that facilitate the swelling of the superficial layer of the tooth polymer, thereby facilitating the diffusion of polymerizable material and enhancing the formation of a high-quality interpenetrating polymer network. Therefore, this treatment leads to a solvent attack on the teeth and produces greater bond strength than other surface treatments. The solubility parameter of this solvent is equivalent to the estimated solubility parameter of an acrylic resin tooth [13]. Similar findings were reported by Krishna et al. [5]. The reason for the increased strength of the dichloromethane and monomer mix compared to that of ethyl acetate might be the presence of an extra monomer.

Previous studies using scanning electron microscopy (SEM) showed that dichloromethane surface treatment created pores on the treated surface with a size of approximately 1 µm after 30 s of surface treatment. This facilitates micromechanical retention and improves bond strength [14]. Unfortunately, to date, no study has been conducted to evaluate the effect of a combined solution of dichloromethane and monomer after the recommended 4 min surface treatment.

It was discovered that the mean SBS in the cyanoacrylate adhesive surface treatment group was considerably lower. This might be the result of a layer that formed at the tooth-denture base interface that prevented chemical bonding from occurring, as well as the development of a strong polymer network as a result of insufficient monomer diffusion across the interface. It has a bond strength of only 68N, which is much lower than the minimum necessary bond strength of 110N. Since denture wearers typically apply a biting force of 90N, both dichloromethane and monomer mix (1:1 volume) and ethyl acetate can be used to strengthen the bond between the debonded acrylic tooth and denture base [15].

Limitations of the study

The present study was an in vitro study with different SBS values in vivo. Therefore, future in vivo studies with larger sample sizes are warranted. Additionally, the type of failure was not evaluated in this study. SEM studies should be conducted to evaluate failure types.

Clinical implications

Chemical treatment with dichloromethane and monomer mix and ethyl acetate can be an effective and simple procedure to enhance the bonding of acrylic teeth with the denture base, thereby reducing repeated denture repair and bonding failures, and enhancing patient satisfaction.

## Conclusions

The current investigation found that Trevalon HI high-impact heat-cure denture base resin provided 6.52% stronger bond strength with cross-linked acrylic teeth than DPI Tuff denture base resin among the two brands of high-impact heat-cure denture base resins processed using the standard technique. The strongest bond was produced by chemically treating cross-linked acrylic teeth with dichloromethane + monomer (1:1 mix by volume) while cyanoacrylate produced the weakest bond, even weaker than that of the control group. Therefore, the ideal combination is thought to be the Trevalon HI high-impact heat-cure denture base resin processed with cross-linked acrylic teeth treated with a dichloromethane and monomer mixture (1:1 by volume).

## References

[1] Sayed ME, Lunkad H, Fageeh I (2021). Comparative evaluation of compressive bond strength between acrylic denture base and teeth with various combinations of mechanical and chemical treatments. Coatings.

[2] Cervino G, Cicciù M, Herford AS, Germanà A, Fiorillo L (2020). Biological and chemo-physical features of denture resins. Materials (Basel).

[3] Costa M, Neves S, Carvalho J, Arantes-Oliveira S, Félix S (2021). In vitro comparative study of microhardness and flexural strength of acrylic resins used in removable dentures. Med Sci Forum.

[4] Takahashi Y, Chai J, Takahashi T, Habu T (2000). Bond strength of denture teeth to denture base resins. Int J Prosthodont.

[5] Krishna VP, Premalatha A, Babu PJ, Raju DS, Kumar MP, Rao DB (2014). Effect of various chemicals on the bond strength of acrylic tooth and denture base -an Invitro comparative study. J Int Oral Health.

[6] Barpal D, Curtis DA, Finzen F, Perry J, Gansky SA (1998). Failure load of acrylic resin denture teeth bonded to high impact acrylic resins. J Prosthet Dent.

[7] Anusavice KJ (2004). Phillip’s Science of Dental Materials. https://www.amazon.in/Phillips-Science-Dental-Materials-11e/dp/0721693873/ref=tmm_hrd_swatch_0?_encoding=UTF8&qid=&sr=.

[8] Sadar L, Dhume S, Maniar N, Prakash Patil J, Rane P, Gandhewar M (2013). Comparative evaluation of shear compressive bond strength between cross-linked acrylic resin denture base and cross-linked acrylic resin teeth with different modifcations of their ridge lap surfaces. J Contemp Dent Pract.

[9] Gupta A, Tewari RK (2016). Evaluation and comparison of transverse and impact strength of different high strength denture base resins. Indian J Dent Res.

[10] Geerts GAVM, Jooste CH (1990). A comparison of the bond strengths of microwave- and water both- cured denture material. J Prosthet Dent.

[11] Raszewski Z, Nowakowska-Toporowska A, Nowakowska D, Więckiewicz W (2021). Update on acrylic resins used in dentistry. Mini Rev Med Chem.

[12] Cunningham JL, Bennington IC (1999). An investigation of the variables which may affect the bond between plastic teeth and denture base resin. J Dent.

[13] Chung RW, Clark RK, Darvell BW (1995). The bonding of cold-cured acrylic resin to acrylic denture teeth. Aust Dent J.

[14] Jain G, Palekar U, Awinashe V, Mishra SK, Kawadkar A, Rahangdale T (2014). The effect of different chemical surface treatments of denture teeth on shear bond strength: a comparative study. J Clin Diagn Res.

[15] Rismanchian M, Bajoghli F, Mostajeran Z, Fazel A, Eshkevari Ps (2009). Effect of implants on maximum bite force in edentulous patients. J Oral Implantol.

